# Qualitative and quantitative characteristics of the extracellular DNA delivered to the nucleus of a living cell

**DOI:** 10.1186/1475-2867-6-23

**Published:** 2006-10-11

**Authors:** Vladimir A Rogachev, Anastasia Likhacheva, Oksana Vratskikh, Lyudmila V Mechetina, Tamara E Sebeleva, Sergei S Bogachev, Leonid A Yakubov, Mikhail A Shurdov

**Affiliations:** 1 Institute of Cytology and Genetics, Siberian Branch of the Russian Academy of Sciences, prosp. Koptyuga 2, Novosibirsk, 630090, Russia; 2 Panagenic International Inc. 2935 Byberry Road, Hatboro, PA, 19040, USA; 3 Novosibirsk State University, ul. Pirogova 2, Novosibirsk, 630090, Russia; 4 OOO Panagen, ul. Choros-Gurkina 29, Gorno-Altaisk, 649000, Russia

## Abstract

**Background:**

The blood plasma and other intertissue fluids usually contain a certain amount of DNA, getting there due to a natural cell death in the organism. Cells of this organism can capture the extracellular DNA, whereupon it is delivered to various cell compartments. It is hypothesized that the extracellular DNA is involved in the transfer of genetic information and its fixation in the genome of recipient cell.

**Results:**

The existence of an active flow of extracellular DNA into the cell is demonstrated using human breast adenocarcinoma (MCF-7) cells as a recipient culture. The qualitative state of the DNA fragments delivered to the main cell compartments (cytoplasm and interchromosomal fraction) was assessed. The extracellular DNA delivered to the cell is characterized quantitatively.

**Conclusion:**

It is demonstrated that the extracellular DNA fragments in several minutes reach the nuclear space, where they are processed so that their linear size increases from about 500 bp to 10,000 bp. The amount of free extracellular DNA fragments simultaneously present in the nuclear space may reach up to 2% of the haploid genome. Using individual DNA fragments with a known molecular weight and sequence as an extracellular DNA, it is found that these fragments degrade instantly in the culture liquid in the absence of a competitor DNA and are delivered into the cell as degradants. When adding a sufficient amount of competitor DNA, the initial undegraded molecules of the DNA fragments with the known molecular weight and sequence are detectable both in the cytoplasm and nuclear space only at the zero point of experiments. The labeled precursor α-dNTP*, added to culture medium, was undetectable inside the cell in all the experiments.

## Background

Few papers today report the interactions between the extracellular DNA and the cell, namely, covering the issues of what DNA and in what form exists in the intercellular space, how the DNA is captured from the pericellular space, what occurs with the DNA in the cytoplasm, and how it behaves in the nuclear space. Even less number of papers allows the overall fate of the extracellular DNA to be traced commencing from the moment it enters the intercellular medium (blood plasma and intertissue fluid) resulting from apoptosis or other cellular processes through its capture by the cell and delivery to internal cell compartments to eventual integration into the recipient genome or otherwise utilization. A developed concept of the turnover of extracellular DNA that would provide a distinct notion of the molecular biological characteristics and functional capabilities of this DNA at each moment of the cycle is yet lacking. Nonetheless, each stage listed has been studied to a certain degree and can be analyzed in the scientific aspect proposed.

Numerous data obtained so far suggest that the intertissue fluids and blood plasma usually always contain DNA, which is a constant component of these tissues [1,2]. Moreover, the DNA content in plasma reflects the functional state of the overall organism. It is reliably known that development of cancers of any etiology is accompanied by a multifold increase in the amount of DNA in intertissue fluids [2,3]. An early work of Ledoux [4] summarizing the previously fragmentary data describes different aspects of the DNA action on the cell and reports numerous facts indicating various effects emerging. Actually, this paper determined all the main directions connected with the effects of extracellular DNA on the cell that are somehow or other studied by the experimenters now.

The first and major fact pointed out in this work is transformation of a certain trait connected with the action of extracellular DNA. The transformation may occur in both the cell culture and the living organism. Later, with the development of corresponding methodical procedures, the papers appeared that confirmed this major observation. Anker et al. [5] demonstrated that treatment of the B lymphocytes incapable of producing antibodies to herpes virus with the supernatant of the T lymphocytes stimulated by the virus or with the DNA extracted from this supernatant induced production of antibodies to herpes virus by these, previously incompetent B lymphocytes. Moreover, the antibodies produced by B lymphocytes carried the allotypic characteristics of the donor T cells. The team of Holmgren [6,7] discovered a horizontal transfer of genetic material via cell engulfment of apoptotic bodies. As was demonstrated, this system required the absence of the cyclin kinase inhibitor p21 to fix and preserve the trait transferred horizontally. The authors assumed that p53 protected the normal cells from replication of the transformed DNA upon cell engulfment of apoptotic bodies via activation of p21. Other researchers [8,9] proposed the term "genometastasis" and the corresponding working hypothesis as well as performed experiments that demonstrated the hypothesized transfer of oncogenes via blood plasma. And finally in the Yakubov's work [10] there was achieved the successful attempt of the reverse transformation of malignant into normal cells using the extracellular fragmented DNA that contained the full set of the genomic sequences.

A contact between the cell and extracellular DNA and penetration of the extracellular material through the cytoplasmic membrane constitute the initial stage in their interaction. The studies performed during the last decade found several routes for DNA penetration into the intracellular space. As was mentioned above, the extracellular DNA can enter the cell with the engulfed apoptotic bodies; moreover, various cell types of the organism are capable of engulfing these bodies. The chromatin of apoptotic bodies enters nuclear compartments and is retained there to 7 days [6,7]. A receptor providing a mediated route for extracellular DNA to enter internal compartments of the eukaryotic cell is described. Two types of receptors localized to the cytoplasmic membrane are responsible for this delivery route of DNA molecules. These are factors with molecular weights of about 30 000 (33 000) and 80 000 (79 000) Da [11-13]. The 30 000-Da receptor is involved in the transport of DNA with a size up to several kilobases pairs. The other receptor binds and conveys inside the cell short double-stranded oligonucleotides. Moreover, these receptors function independently and do not compete for the substrate, and their content per cell amounts to approximately 10^4 ^molecules [12]. Note that DNA penetrates the cell during a very short time (several seconds to several minutes), as was demonstrated in early works [4,14]. The studies in question confirm that this process is not connected with DNA degradation and its further resynthesis inside the cell [15]. The studies guided by Yakubov [14,16] discovered that oligonucleotides were transported to the nucleus with the same proteins to which they bound on the cell surface and that were responsible for oligonucleotide transfer into internal cell compartments. It is also necessary to mention the transport of large DNA molecules into organelles. It was found that extracellular DNA is imported into plant mitochondria, where it is accumulated and is either transcribed or used as a substrate for repair synthesis [17].

In this work, we focused the attention on research into the behavior of fragmented DNA when it enters the cell compartments of MCF-7 cells.

## Results

### Distribution of extracellular DNA with a length of 500 bp, represented by a pool of fragments comprising the complete human genome, over cell compartments

#### Figures necessary for quantitative assessment of experimental results

The counts of human breast adenocarcinoma cells MCF-7 per experimental point were ***0.7 *× *10*^6^**.

According to the protocol for ***MCF-7, 10^7 ^***cells contain ***60 μg *DNA**.

One cell contains ***6 picogram ***of DNA.

The total DNA amount at experimental point was ***4.2 μg***.

The amounts of labeled DNA added to experimental point are listed in Table 1. Radioactivity counts of the added DNA (***cpm***) are shown in Table 1.

**Table 1:** General characterization of the amounts of material taken in the experiments

	**Experiment II**		**Experiment III**		**Experiment IV**	
	
	**μg**	**cpm**	**μg**	**cpm**	**μg**	**cpm**
DNA* amount (μg and cpm) per point	1.4	1.08 × 10^7^	3.6	2.04 × 10^7^	2.5	1.8 × 10^7^
α-dATP*amount (cpm) per point		0.9 × 10^7^		2.0 × 10^7^		1.4 × 10^7^

Radioactivity counts of α-dNTP* per experimental point (***cpm***) amounted to the values listed in Table 1.

The haploid human genome contains 3.3 × 10^9 ^bp.

The initial size of labeled DNA fragments added to the medium amounted to 300 bp.

Two stages of the culture growth (exponential phase and the phase of contact inhibition) and two types of electrophoretic assay (under native and denaturing conditions) were chosen for analyzing the behavior of extracellular DNA. Qualitative characteristics and quantitative parameters of extracellular DNA behavior in the cell were analyzed.

### Qualitative behavior characteristics of labeled material (native conditions). Exponential phase of cell growth at the moment DNA and precursor are added to culture medium (Fig. 1)

**Figure 1:**
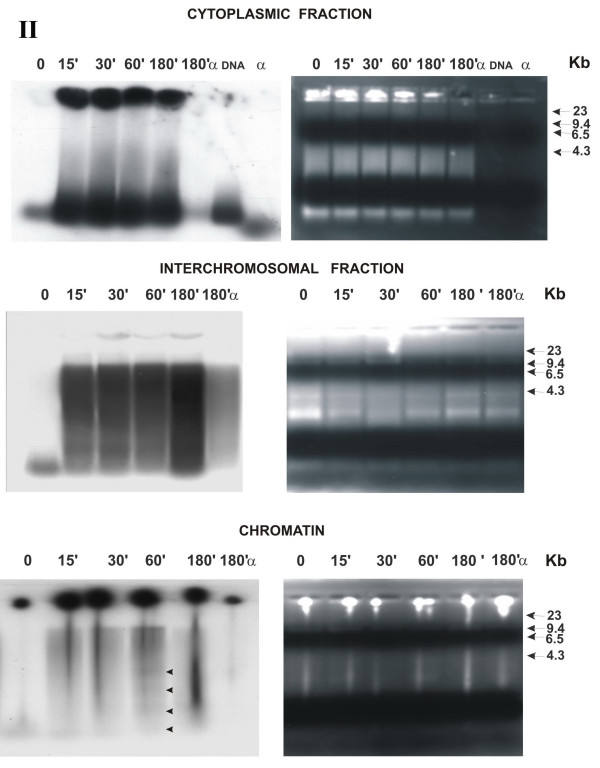
Distribution of fragmented extracellular human DNA over various cell compartments of MCF-7 cell culture depending on the time it was present in the culture medium **(electrophoresis under native conditions, logarithmic stage of cell culture growth)**. Agarose blocks stained with ethidium bromide are shown rightward; X-ray patterns of the same blocks after drying, leftward. Numbers above the blocks indicate the time of cell culture incubation with α^32^P-labeled extracellular DNA and α-dATP*; numbers to the right of the blocks, markers of molecular weight (Kb); α, initial α-dATP* precursor; II, number of experiment. Arrows denote fragments of processed DNA localized to the interchromosomal space.

All figures contain electrophoretic patterns (right) and the corresponding X-ray patterns obtained by exposure to the same agarose blocks upon drying (left).

#### Cytoplasmic fraction (upper group of blocks)

DNA virtually immediately (detectable at the zero point; see Materials and Methods) enters the cell cytoplasm. The low-molecular-weight fraction, corresponding in its mobility to the initial labeled material, is seen at the zero point. The label is absent at the start of the zero point. In the rest samples, a considerable amount of the labeled material is present at the start. This suggests that DNA forms high-molecular-weight complexes with either certain components of the cytoplasm or buffer components, or with both. An evident jellyfish of precipitating DNA is absent. All samples display the DNA fraction similar in its mobility to the mobility of initial DNA added to the medium. This means that the freely migrating part of the DNA delivered to the cytoplasm is not metabolized. The other part of DNA, which remains at the start, either underwent certain transformations or is a component of high-molecular-weight complexes. We do not analyze the qualitative start of the latter part of labeled material.

#### Interchromosomal fraction (middle group of blocks)

DNA virtually instantly penetrates into the interchromosomal fraction of the nucleus. At the zero point, a low-molecular-weight fraction is seen, which is similar in its mobility to the initial labeled material. Label is absent at the start of zero point and at the starts of the rest samples. The overall high-molecular-weight fraction forms a lenticular pellet during centrifugation, which is undetectable in the nuclear sap that contains interchromosomal DNA. A change in DNA mobility is observed in all the samples except for zero point. The initial DNA forms a smear-like high-molecular-weight pool rising from the lower part to the high-molecular-weight zone of the agarose block, reaching the size of about 10 kbp.

#### Chromatin (lower group of blocks)

Labeled material virtually instantly (detectable already at the zero point; see Materials and Methods) penetrates into the nucleus and is revealed in the fraction of nuclear chromatin. The separation zone of gel displays a pronounced ladder of the fragments with sizes multiple to the initial DNA (black arrows). (This labeled material appears due to an insignificant contamination with the interchromosomal fraction which contains the label with a high specific activity.)

#### α-dATP* (in all blocks, designated 180α)

αdATP* is absent in the cytoplasmic fraction. However, a labeled fragment coinciding in its size with the DNA used in experiment is detected. It forms in the interchromosomal fraction a smear-like DNA pool morphologically similar to that for the analogous fraction with labeled DNA. The label is detectable in the chromatin after 180 min (Table 2). The rest points for αdATP* were not controlled. The amount of label taken in the culture media is approximately equal for labeled DNA and αdATP* (see tables). However, the amount of labeled material detected at this experimental point for αdATP* is approximately sixfold lower compared with that for DNA.

**Table 2:** Absolute counts (AC, cpm) and percent of the α-dATP*-labeled material added to culture medium

α-dATP* 180 min	**Experiment II Exponential phase**	**Experiment III Exponential phase**	**Experiment IV Cell monolayer**
Cytoplasmic fraction (cpm)	94 775	128 271	348 924
% of the amount added to medium	1.1%	0.64%	2.49%
Interchromosomal fraction (cpm)	31 328	13 886	128 212
% of the amount added to medium	0.35%	0.07%	0.88%
Chromatin (cpm)	5 915	14 689	32 516
% of the amount added to medium	0.066%	0.07%	0.22%

### Qualitative behavior characteristics of labeled material (denaturing conditions). Exponential phase of cell growth at the moment DNA and precursor are added to culture medium (Fig. 2)

**Figure 2:**
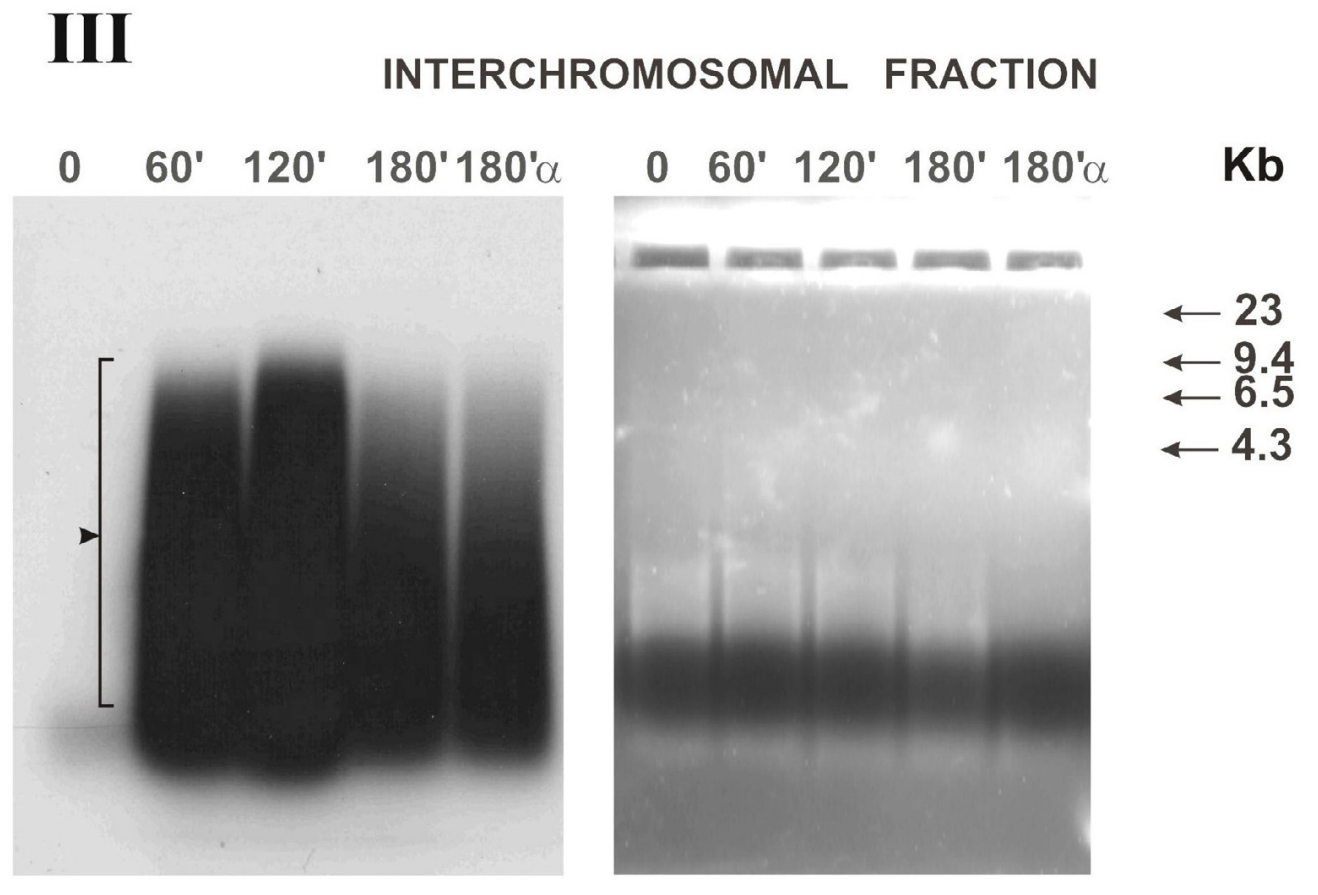
Distribution of fragmented extracellular human DNA over interchromosomal fractions of MCF-7 cell culture depending on the time it was present in the culture medium **(electrophoresis under denaturing conditions, logarithmic stage of cell culture growth)**. Agarose block stained with ethidium bromide are shown rightward; X-ray patterns of the same block after drying, leftward. Numbers above the block indicate the time of cell culture incubation with α^32^P-labeled extracellular DNA and α-dATP*; numbers to the right of the blocks, markers of molecular weight (Kb); α, initial α-dATP* precursor; III, number of experiment. Arrow denotes polymeric DNA form, retaining its size under denaturing conditions.

A series of denaturing electrophoreses was performed to clarify whether the increase in linear sizes of extracellular DNA was related to joining of the fragments into a continuous chain. We assumed that if the slowing of labeled material migration were connected with formation of stable complexes, the denaturing conditions would detect the monomers of the complexes.

#### Cytoplasmic fraction and chromatin (data not shown)

The overall pattern remained the same compared with the experiments under native conditions.

#### Interchromosomal fraction (Fig. 2)

The overall pattern remained virtually the same. Polymeric DNA fraction reached a size of 10 kbp. Distinct banding pattern disappeared.

#### α-dATP* (in all blocks, designated 180α)

The behavior pattern is similar to that under native conditions. The initial α-dATP form is undetectable in the cell.

### Qualitative behavior characteristics of labeled material (denaturing conditions). Cell monolayer upon addition of labeled DNA to the medium. Phase of contact inhibition (Fig. 3)

**Figure 3:**
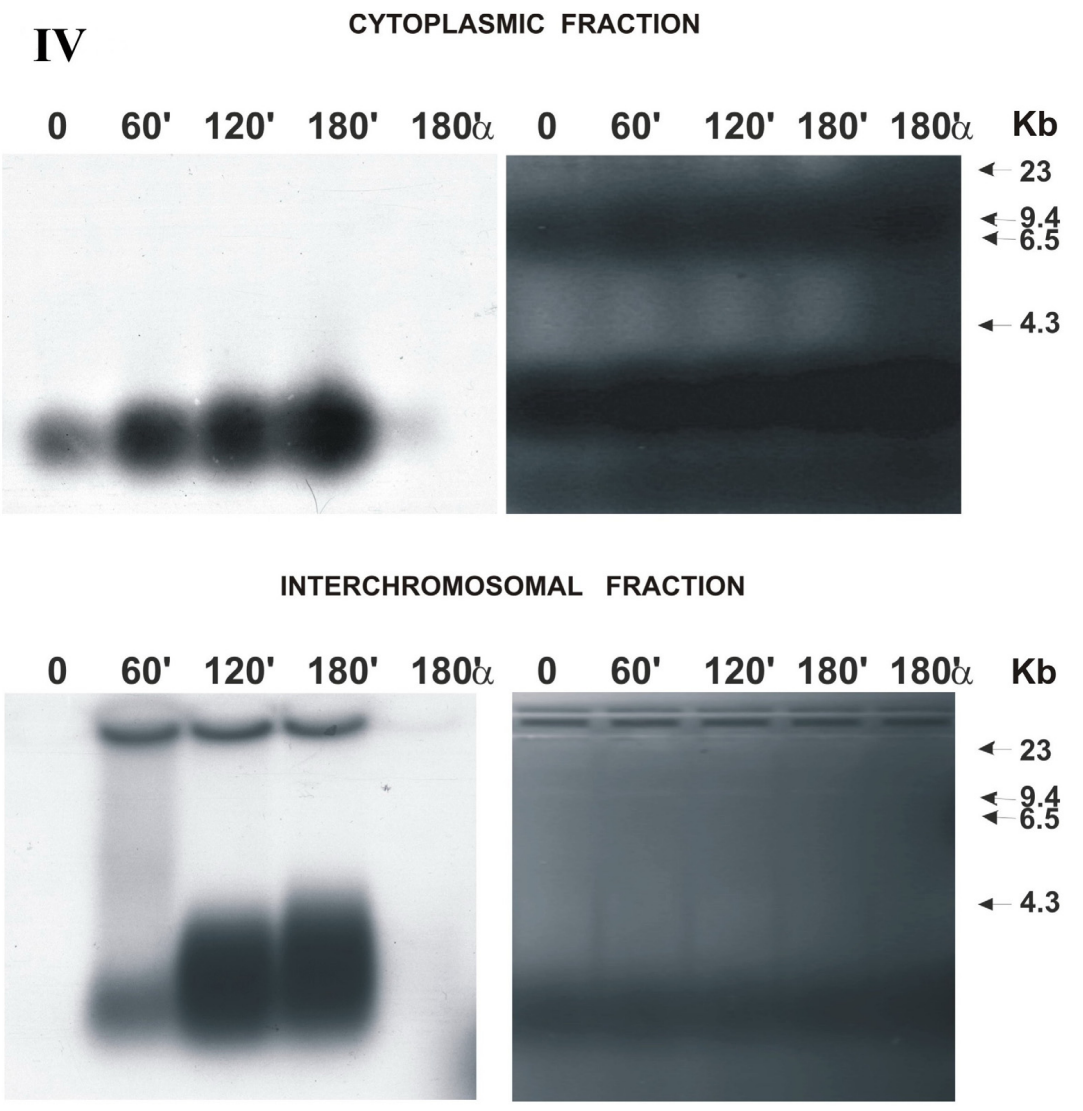
Distribution of fragmented extracellular human DNA over various cell compartments of MCF-7 cell culture depending on the time it was present in the culture medium **(electrophoresis under denaturing conditions, stage of contact growth inhibition)**. Agarose blocks stained with ethidium bromide are shown rightward; X-ray patterns of the same blocks after drying, leftward. Numbers above the blocks indicate the time of cell culture incubation with α^32^P-labeled extracellular DNA and α-dATP*; numbers to the right of the blocks, markers of molecular weight (Kb); IV, number of experiment. Accumulation of labeled material in the cytoplasm and increase in the linear size of initial fragmented DNA in the interchromosomal space are distinctly seen.

All the processes were considerably less intensive compared with the stage of cell culture logarithmic growth. The cytoplasmic fraction displays a distinct label accumulation. In the interchromosomal fraction, the linear size of the initial DNA fragment increases successively with the increase in the time of exposure to labeled DNA. However, this pattern becomes distinct only at the point of 120 min. The zero point contains no label; the point of 60 min displays the DNA fragment of initial size. Similar to previous experiments, α-dATP* as a monomer is absent in all the cell compartments.

The agarose blocks stained with ethidium bromide demonstrate that the nucleic acid material is present in the samples; however, it is nonuniform depending on the compartment wherefrom the sample originates.

#### Cytological examination (Fig. 4)

**Figure 4:**
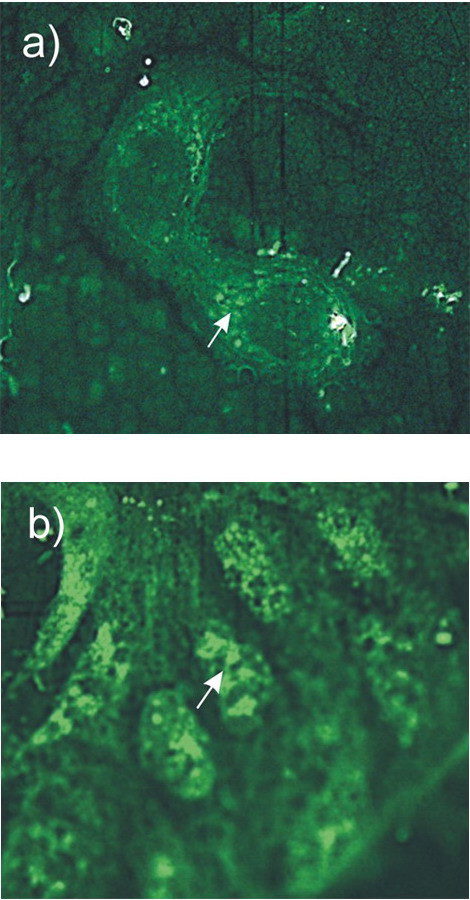
Cytological examination of the labeled DNA distribution in MCF-7 cells. (a) Several minutes incubation of MCF-7 cells with FITC-labeled fragmented human DNA. The main part of the label is localized to the cytoplasm. (b) 14-h incubation of MCF-7 cells with FITC-labeled fragmented human DNA. The label is concentrated in the nucleus and is undetectable in the cytoplasm.

A large amount of labeled material is observed in the cytoplasm after a several-minute exposure of MCF-7 cells to FITC-labeled DNA (Fig. 4a). After a 14-h exposure, all label concentrates in the cell nuclei (Fig. 4b). The labeled material is present in the cytoplasm as vacuolar vesicles. In the nuclei, the labeled material is also distributed in a form of nodules or vesicles.

### Quantitative behavior characteristics of labeled material

**Figure 5:**
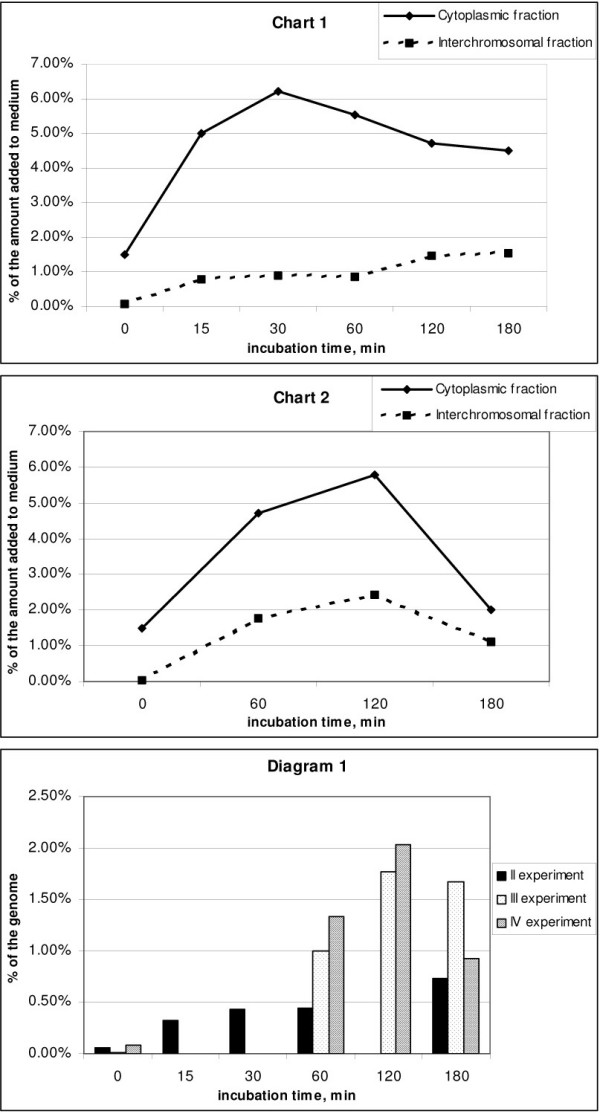
The dynamics of internalization of the labeled extracellular material into cell compartments. Chart 1. Exponential phase (The values averaged over experiments II and III). % of the amount added to medium in the cytoplasmic and interchromosomal fractions. Chart 2. Cell monolayer (IV experiment). % of the amount added to medium in the cytoplasmic and interchromosomal fractions. Diagram 1. Amount labeled material that is detected in the interchromosomal fraction (% of the genome). Experiments II, III and IV.

The quantitative estimates made based on comparison of the counts at experimental points for various cell compartments are listed in below tables. The dynamics of accumulation of the labeled material in the cell compartments is presented on the Fig. 5 (Charts 1 and 2). Diagram 1 from Fig. 5 illustrates the dynamics of accumulation and the maximum amount of the extracellular DNA that is revealed in the free form in the nucleus (interchromosomal fraction). Note that the labeled material during all the DNA-purification procedures is present in the absolute amount; therefore, it is appropriate to compare and relate it to the label initially introduced into the medium.

### Distribution over cell compartments of the extracellular DNA represented by individual fragments with blunt and 3'-sticky ends in the presence of competitor DNA and without it

When analyzing the changes in the fragmented genomic DNA after it entered various cell compartments, we discovered the fact of processing of the extracellular DNA delivered to the nucleus and deposited with the interchromosomal space. The linear DNA size during processing increased to about 10000 bp and was retained under denaturing conditions. We hypothesized that the increase in question may result from either ligation of various fragments or a certain variant of synthesis – involving fragments as primers for polymerization on a chromosome template or using the labeled material hydrolyzed to monomers. Note that in no experiment we observed the labeled material corresponding to the precursor monomer (α-dATP*). To test what possibility was actually realized, we prepared two DNA substrates and incubated MCF-7 cells with these DNAs for various times. One substrate was the plasmid Carnegie 20-λ1.4(× 8), comprising the vector Carnegie 20 and eight copies of *D. melanogaster *1.4-kbp M/SAR *Sal*GI fragment [18] [EMBL, GenBank, DDBJ: Z37541], hydrolyzed with *Sal*GI restriction endonuclease. This hydrolysate was labeled with α-dATP* using Klenow fragment in the presence of the three rest cold triphosphates and completed with cold dATP to form blunt ends. The other substrate was represented by 506- and 230-bp PCR fragments of human caspase-3 gene [GenBank: NM_004346]. Both fragments were labeled directly during PCR. As is known, the thermophilic polymerase attaches one surplus nucleotide, predominantly dA, at the 3'-ends of the fragments synthesized. We tested the feasibility of ligation of both the first and second substrates in an *in vitro *system. Carnegie 20-λ1.4(× 8)*Sal*GI ligated with the efficiency characteristic of the fragments with blunt ends. As for the 506- and 230-bp fragments, we succeeded in detecting ligation products only upon blunting the 3'-ends of these fragments with S1 nuclease (data not shown). These manipulations produced two DNA substrates – the substrate with blunt ends and the substrate with one-nucleotide tagged 3'-ends. Using these substrates, we planned to determine which of the processes occurred – ligation or synthesis. In the first case, we would observe a ladder of bands multiple to the lengths of the substrate fragments. In the second case, we should anticipate a smeared pool of DNA with increasing lengths of fragments. In addition, we tried to answer the question on whether the physicochemical structure of the fragments' ends was important. Two blocks of electrophoretic patterns, described below, resulted from these experiments. MCF-7 cell culture at the stage of logarithmic growth and native condition of DNA isolation and electrophoresis were used in both experiments. The cells were incubated with substrates without and in the presence of competitor DNA. Salmon sperm DNA at a concentration of 200 μg/ml was used as a competitor. About 3 μg of the labeled substrate was taken per one experimental point.

### Intracellular localization of and morphological changes in Carnegie 20-λ1.4(× 8)SalGI substrate (labeled with α-dATP*) during incubation with MCF-7 cells without competitor DNA

The band morphology changed in the medium immediately at the point of 30 min. The initial labeled material was detectable neither after 30 min nor at any other time point in any cell compartment. (Experiment V).

**Figure 6:**
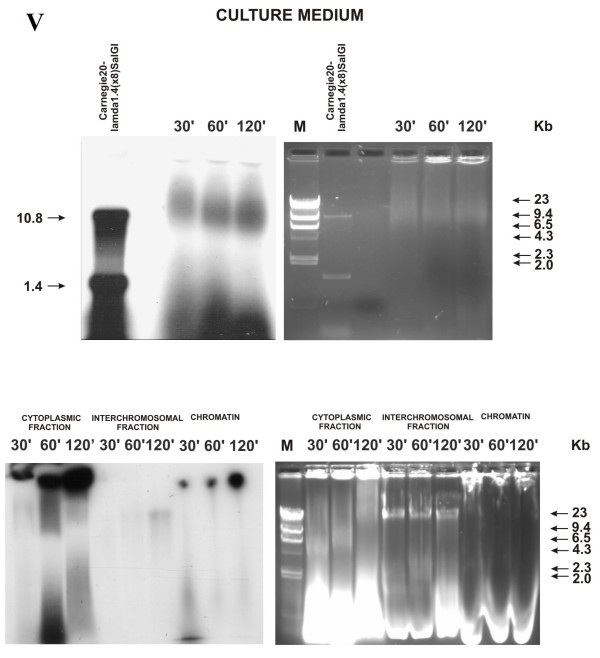
Distribution of two individual DNA fragments with lengths of 10.4 and 1.4 kb over various cell compartments of MCF-7 cell culture depending on the time they were present in the culture medium **(electrophoresis under native conditions, logarithmic stage of cell culture growth, absence of salmon sperm DNA as competitor, and blunt ends)**. Agarose blocks stained with ethidium bromide are shown rightward; X-ray patterns of the same blocks after drying, leftward. Numbers above the blocks indicate the time of cell culture incubation with α^32^P-labeled fragments; numbers to the left and right of the blocks, markers of molecular weight (Kb); V, number of experiment. Degradation of the initial DNA fragments is observed already in the culture medium. Similar to human DNA, the heterologous DNA enters general cell compartments – cytoplasm and nucleus.

In the medium, the labeled fragments changed their mobility, forming smeared spots of the processed heavy and light fragments (Fig. 6). Both fragments (in an altered form) initially displayed a decreased mobility in the gel. The heavy fragment at the last point (120 min) migrated similarly to the initial fragment but was visible as a smeared spot. The short fragment degraded to a length of approximately 200 bp by 120 min of incubation.

The DNA processed similarly is found in the cytoplasmic fraction. We failed to detect any signs of discrete fragments in the interchromosomal fraction. Similarly to the experiments of the first section, the chromatin fraction contained a considerable amount of the labeled material. The overall pattern displayed by the labeled material remained the same as that obtained during the first set of experiments. An increase in the amount of labeled material in both compartments of the nucleus with the incubation time of cells with DNA substrate was evident.

Note that two individual fragments were used as a substrate. However, in no point of experiment we detected two initial labeled fragments. Presumably, all DNA fragments in the culture medium degraded during incubation.

### The effect of competitor DNA on physical parameters of individual fragments during joint incubation with MCF-7 cells

**Figure 7:**
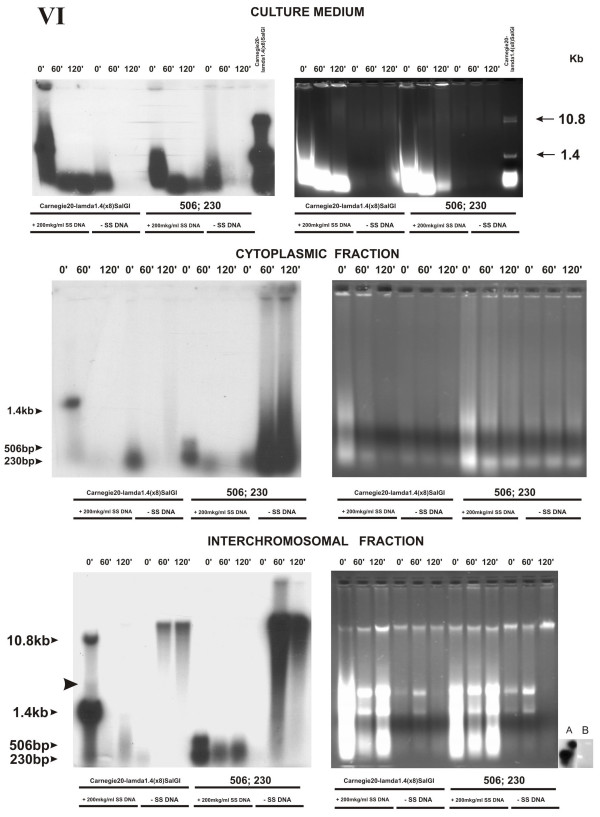
Distribution of individual DNA fragments with lengths of 10.4 and 1.4 kb with blunt ends (designated as Carnegie 20-λp1.4(× 8)*Sal*GI) either in the presence of salmon sperm DNA (SS DNA) as a competitor or without it and of individual PCR fragments of 230 and 506 bp with tag 3'-ends synthesized from human caspase-5 gene template (designated as 230 and 506) under the same conditions over various cell compartments of MCF-7 cell culture depending on the time they were present in the culture medium **(electrophoresis under native conditions, logarithmic stage of cell culture growth)**. Agarose blocks stained with ethidium bromide are shown rightward; X-ray patterns of the same blocks after drying, leftward. Numbers above the blocks indicate the time of cell culture incubation with α^32^P-labeled fragments; numbers to the left and right of the blocks, markers of molecular weight (Kb); VI, number of experiment. (A) X-ray pattern of the PCR labeled 506- and 230-bp fragments. (B) The PCR 506- and 230-bp fragments fractionated by agarose electrophoresis, stained with ethidium bromide. Arrow indicates the dimer of 1.4 kb fragment.

To determine the effect of competitor DNA on physical parameters of the substrate (Experiment VI), we incubated two types of fragments – with blunt ends (Carnegie 20-λ1.4(× 8)*Sal*GI) and with tag 3'-ends (506- and 230-bp) – with MCF-7 culture in the presence of 200 μg/ml salmon sperm DNA (Fig. 7). Since the initial labeled material in the previous experiment was undetectable already at the point of 30 min, we took the zero point (0 min) as the first experimental point in this experiment. Fig. 7 demonstrates the obtained distribution pattern of the labeled fragments and changes in their parameters. Similarly to the previous experiment, the labeled material in the absence of competitor DNA in the medium commenced degrading already at the zero point independently of the substrate. The size of completely degraded fragments amounted to about 200 bp.

As for the variant with 200 μg/ml of salmon sperm DNA in the medium, intermediate degradation phases were observed at the zero point. The light fragments (1.4 kb, 506, and 230 bp) remained intact. The heavy fragment (Carnegie 20-λ1.4(× 8)*Sal*GI) degraded completely in the medium already at the zero point. With further increase in incubation time, all DNA in the medium degrade to fragments with a length of approximately 200 bp, which are then engulfed by the cells. Analysis of the cytoplasmic and, especially, interchromosomal fractions brought the following surprising phenomenon. Despite that the heavy fragment was undetectable in the medium already at the point of 0 min, it was present at the same time moment in the interchromosomal fraction. Together with heavy fragment, the rest labeled fragments – 1.4 kb, 506 bp, and 230 bp – were present in an intact state at the zero point in both the cytoplasm and interchromosomal space. Presumably, the presence of competitor DNA blocks the initial nuclease activities, thereby sparing some time for a certain amount of the native fragments to enter the cell before any contact with the nucleases in the medium; this took one-two minutes (the time required to add lysing buffer and start gradient centrifugation). At the point of 0 min, both native monomers and the fragment multiple to two 1.4-kbp monomers were observed for Carnegie 20-λ1.4(× 8)*Sal*GI substrate in the presence of competitor DNA (Fig. 7, interchromosomal fraction). However, we did not find any fragments with lower mobility that were multiple to the fragments of 506 and 230 bp labeled with the same intensity. This suggests that ligation occurs inside the nucleus and that the structure of the fragment's ends is important for this process. In the case of longer incubation times, the already degraded fragments whose lengths are about 200 bp are delivered into the cell. Presumably, ligation of heterogeneous degrading fragments leads to the smeared tail of the labeled material. Staining of gels with ethidium bromide demonstrated that a considerable amount of competitor DNA was also delivered to the interchromosomal compartment of the nucleus in addition to the tracer fragment.

## Discussion

### Qualitative changes in the extracellular DNA delivered to cell compartments

The main goal of this work was to attempt tracing the mere possibility and the changes that could occur in the extracellular exogenous DNA transported to various cell compartments. We assumed that the DNA fragmented to a size multiple to 1–10 nucleosome units and composed of the fragments representing the complete human genome would be the most "physiological" for its recognition by DNA delivery and utilization mechanisms. This DNA size corresponds to the size of the apoptotic DNA present in the blood plasma. As was mentioned repeatedly, many examples reporting delivery of DNA of various compositional complexities to the cell are described. We believed possible to trace the route of DNA and the changes it underwent when entering the cytoplasm and nucleus via a natural delivery mechanism used by the cell. Two sets of experiments were performed. In the first set, the genomic DNA fragmented to a length of about 500 bp and labeled with α^32^P* by nick translation, was used. In the second set, individual labeled fragments were used for analysis of the events taking place during transportation of nucleic acids into the cell. Various phenomena were found indicating that the extracellular DNA that entered the cell became an active component of the processes occurring in the cell.

The first set of experiments gave an unanticipated result that, first, α-dATP* was conveyed to all the cell compartments not as a monomer, but within DNA fragments of about 200 bp long. Further, this DNA is involved in all the processes similar to the DNA added to the medium. In all respects, the activity of α-dATP* utilization is severalfold less intensive compared with the utilization of labeled extracellular DNA. Then it was found that extracellular DNA was virtually immediately (less than during 1 min if the cells were actively dividing) delivered to all the cell compartments analyzed (cytoplasm and nuclear space) and label appears in the chromatin. In the cytoplasm of actively dividing cells, approximately equal amount of labeled DNA was present at all the experimental points except for zero point (Fig. 5, Chart 1). The label accumulated in the nuclear compartments depending on the time of exposure to labeled DNA present in the culture liquid. DNA was present in the interchromosomal space of the nucleus as fragments of various lengths. The size of the fragments at the zero point in the actively dividing cells corresponds to the size of extracellular DNA added to the medium. In all the rest variants, the linear DNA size increased to approximately 10 kb. Moreover, the "ladder" of the bands detected (Fig. 1, left lower block), suggests that a part of the fragments were ligated with one another. In cells of the culture at the stage of contact inhibition, DNA processing (denaturing conditions) displayed a distinct dynamics (Fig. 3). Presumably, the metabolic processes at this stage of cell culture growth are slowed down, and we succeeded in detecting the dynamic stage of the processes of accumulating the labeled material in the cytoplasm (separation zone of gel; freely migrating DNA) and forming multimers of the initial DNA fragments in the nucleus. Increase in the linear size of the DNA molecules delivered to the nuclear space may result from the specific DNA ligase IV activity, involved in repair events [19].

In the second set of the experiments, it was found, first, that any DNA added to the culture medium of MCF-7 cells was degraded virtually immediately. Only the fraction of tracer DNA, which was protected from nuclease activities by a high concentration of competitor DNA and thereby succeeded in binding to surface cytoplasmic factors and entering cell compartments,

When using a high concentration of competitor DNA, a certain amount of initial fragments is virtually immediately delivered to cell compartments in an intact state. At zero point when using the fragments with blunt ends and excess of competitor DNA, we detected in the interchromosomal fraction emergence of a weak but distinct fragment that could be a dimer produced by ligation of the 1.4-kbp fragment (Fig. 7, left lower block; indicated by an arrow). No discrete bands with a low electrophoretic mobility were detected for the variant with tag 3'-ends.

Cytological examination of the behavior of extracellular DNA during its contact with cells demonstrated that the labeled material appeared in the cytoplasm within vacuoles already after several minutes. The label was virtually undetectable in the nuclear space. The entire label was localized to the nuclear compartments after a 14-h incubation. In the nucleus, the labeled material was located in a form of conglomerates and displayed an intensive fluorescence. It is known that pinocytosis is a general mechanism for engulfing any extracellular material. The corresponding hypothesis states that the exchange of macromolecules or their fragments between the cell and environment is bidirectional. This means the possibility of an "intravital" exchange of genetic information between individual cells of the organism via releasing the DNA fragments that appeared as a result of extracellular DNA turnover by reverse pinocytosis. This concept may entail another hypothesis assuming that a certain part of the DNA molecules present in the cell comprises the cell DNA carrying the information about metabolism of this cell itself, whereas the other part is the population of molecules that could have been conveyed from the ambient medium. The latter molecules may carry the information about the overall organism, the organism as a species (and as any other entity), and have little in common with the information about the overall metabolism of this particular cell [4]. A continuous sorting of such molecules may form the informational mechanism that transfer the data about the general state of the organism to individual cells.

### Assessing amounts of extracellular DNA detected in cell compartments

The amounts of extracellular DNA delivered to cell compartments are estimated as tenths of a percent to percents of the DNA present in the culture medium. A simple estimation of the delivery dynamics of extracellular DNA to the cell space displays the following trends.

The label is quickly accumulated in the cytoplasm (Table 3; Fig. 5, Charts 1, 2). The maximal DNA amount is detected between 60 and 120 min, followed by a decrease by 180 min.

**Table 3:** Quantitative characteristics of the labeled material content in various cell compartments depending on the time of its incubation with cells

**Incubation time**	**0 min**			**15 min**			**30 min**			**60 min**			**120 min**			**180 min**		
**Experiment no.**	**II**	**III**	**IV**	**II**	**III**	**IV**	**II**	**III**	**IV**	**II**	**III**	**IV**	**II**	**III**	**IV**	**II**	**III**	**IV**

Cytoplasmic fraction (absolute counts, cpm)	74 883	470 559	275 481	542 304			674 216			677 588	980 421	854 765		960 000	1 060 674	518 282	863 345	368 101
% of the amount added to medium	0.7%	2.3%	1.5%	5.0%			6.2%			6.3%	4.8%	4.7%		4.7%	5.8%	4.8%	4.2%	2.0%
Amount in μg according to %	0.0098	0.08	0.0375	0.07			0.087			0.088	0.17	0.118		0.17	0.145	0.67	0.15	0.05
Interchromosomal fraction (absolute counts, cpm)	11 460	3060	7330	83 662			95 821			97 147	159 378	322 008		299 963	445 426	177 814	282 826	203 421
% of the amount added to medium	0.1%	0.015%	0.04%	0.77%			0.89%			0.9%	0.78%	1.75%		1.47%	2.42%	1.65%	1.39%	1.1%
Amount in μg according to %	0.0014	0.0005	0.0025	0.01			0.013			0.0135	0.03	0.04		0.053	0.061	0.023	0.05	0.028
% of the genome	0.066%	0.016%	0.083%	0.33%			0.43%			0.45%	1.0%	1.33%		1.77%	2.03%	0.73%	1.67%	0.92%
Chromatin (absolute counts, cpm)	8089	3691	2248	45 812			47 684			49 394	296 771	123 390		242 836	157 124	37 620	244 937	118 285
% of the amount added to medium	0.075%	0.018%	0.012%	0.4%			0.44%			0.46%	1.45%	0.67%		1.19%	0.85%	0.35%	1.2%	0.64%
Amount in μg according to %	0.0011	0.00065	0.0003	0.0056			0.006			0.0064	0.05	0.0168		0.0428	0.021	0.0042	0.043	0.016

The increase in DNA amount in the interchromosomal space up to the point of 120 min followed either by a decrease. We estimated percentage of the DNA localized to the interchromosomal space with respect to the haploid genome. It appeared that in the case of the indicated DNA content in the culture medium, 2 000 000 to 66 000 000 bp (or 0.66–2% of haploid genome) is accumulated in the interchromosomal space (Fig. 5, Diagram 1). As it was mentioned above, the DNA is represented by 500-bp fragments, a part of which is joined in concatemers. This high number of free double-stranded ends is likely to activate drastically the repair and recombination machinery of the cell and may cause an active integration of extrachromosomal material into the chromatin.

The plot of labeled material accumulation in the chromatin is smoother. The labeled material is accumulated over 60 min followed by reaching the plateau that continues to the last experimental point of 180 min in all experiments. Presumably, a considerable amount of the labeled material detected in the chromatin is connected not with the integration, but with the re-utilization of labeled material. Nonetheless, our data on rescue of mutation in caspase-3 gene [20] may suggest that a certain part of the extrachromosomal fragments integrate into the genome in a legitimate manner via homologous recombination.

The data reported suggest that a certain amount of DNA under the specified experimental conditions (MCF-7 cell culture, DNA concentration, number of cells, and reaction media) is delivered to the nucleus, constantly presents in the interchromosomal space, and possibly integrates into the genome. In the interchromosomal space, DNA undergoes alterations that increase its linear size to about 10 kbp.

Analyzing the events that occur when the extracellular DNA appears in the nucleus, the following questions are of the greatest importance. Is the DNA flow into the cell constant and does it appear to be a part of the general cell mechanism of the extracellular DNA utilization? Does the DNA delivered to the nucleus induce recombination process? What is the intensity of this process and does this intensity depend on the amount of DNA delivered to the nucleus? What factors influence the ratio of legitimate to nonlegitimate recombination?

We assume that the extracellular DNA is an important component of the molecular processes running in the organism.

## Conclusion

A constant flow of extracellular DNA into the cell exists in the human breast adenocarcinoma MCF-7 cells. The fragments of extracellular DNA in several minutes reach the nuclear space, where they are processed so that their linear size increases from about 500 bp to 10,000 bp. The amount of extracellular DNA fragments simultaneously present in the nuclear space may reach up to 2% of the haploid genome. In the absence of competitor DNA, these DNA fragments degrade instantly in the culture liquid and are delivered into the cell as degradants. When adding a sufficient amount of competitor DNA, the initial undegraded molecules of the DNA fragments are detectable both in the cytoplasm and nuclear space only at the zero point of experiments. α-dNTP* in the initial form (added to culture medium) is undetectable in the intracellular compartments.

## Methods

### Cell culture

Human breast adenocarcinoma (MCF-7) cells were cultivated in the RPMI 1640 medium supplemented with 10 mM L-glutamine and 50 μg/ml streptomycin (Sigma, USA) at 37°C in the presence of 5% fetal bovine serum (FBS; Biolot, Russia) in an atmosphere of 5% CO_2 _to a density of 0.7 × 10^7 ^cells per 4 wells of a 24-well plate.

### Preparation of DNA and precursor

Human placental DNA (10 μg) fragmented to 500 bp was labeled by nick translation in the presence of Klenow fragment, three cold, and one hot dNTPs. The unincorporated precursors were removed by double precipitation with isopropanol according to the protocol described by Glover [21]. The yield of labeled DNA upon purification amounted to 7–15 μg. DNA was dissolved in an appropriate volume of distilled water and added to wells of the plate (no more than 20 μl per well). Each well contained 250–350 μl of the medium. A 1-μl aliquot was taken from the working volume to determine radioactivity.

The aliquot of α-dNTP* was diluted in the appropriate volume of water, and 1 μl of the diluted triphosphate was sampled to count radioactivity. An approximately similar amount of α relative to DNA (with respect to radioactive count and volume) was added per each point.

The amounts of DNA (μg and cpm) and α-dNTP* (cpm) for the three experiments are listed in Table 1.

For cytological examination, DNA was labeled with the modified precursor containing FITC fluorochrome.

Restriction fragments were labeled at the sticky end formed by hydrolysis with *Sal*GI restriction endonuclease in the presence of Klenow fragment, three cold triphosphates, and hot dATP*. Upon attachment of cold dATP to make the ends blunt, DNA was purified from the unincorporated precursors by double precipitation with isopropanol from 0.3 M NaAc.

DNA of the PCR fragments of 506 and 230 bp was labeled during PCR using specific primers [20].

### Treating cells with fragmented DNA, preparing cell extracts, and assaying DNA in cell compartments

The labeled DNA treated as described above was added to the culture medium into the well where cells were cultivated. Cells were incubated with DNA at 37°C for the time required in an air thermostat. The following incubation times were chosen: 0 (labeled DNA was added to the medium in a titrator placed on ice; the medium was immediately sampled and supplemented with Triton X-100), 15, 30, 60, 120, and 180 min with variations in different experiments. The incubation time for α-dATP* was 180 min. The DNA amounts in the incubation medium per point are listed in Table 1.

After incubation, the plate was placed on ice. The medium was quantitatively sampled, and buffer A containing 2 mM CaCl_2 _and 0.5% Triton X-100 [22], was immediately added to cells. Each well was supplemented with 250 μl of lysing buffer, being 1 ml per point. Cells were kept with lysing buffer on ice for 10 min. The lysed cells were resuspended several times and loaded onto 1 ml of sucrose gradient in buffer A with 2 mM CaCl_2 _and centrifuged in conic tubes (25 ml) in a bucket rotor using a K23 centrifuge at 600 × *g *(2000 rpm) for 20 min. The supernatant, containing cytoplasmic fraction, was collected in a separate tube to precipitate with isopropanol from 0.3 M NaAc. The pellet of nuclei was washed twice with 250 μl of lysing buffer (buffer A containing 2 mM CaCl_2 _and 0.5% Triton X-100). After each washing, the preparation was centrifuged for 10 min in a bucket rotor in a K23 centrifuge at 600 × *g *(2000 rpm). The nuclei were resuspended in 500 μl of buffer A containing 2 mM CaCl_2_, examined cytologically, and transferred to the tubes of a JA-21 rotor of a J2–21 centrifuge. The suspension of nuclei was supplemented with 2 M NaCl and 1% SDS. Nuclear lysate was incubated at 65°C for 15 min until a complete clarification of the reaction mixture without any shaking. The nuclear lysate was then centrifuged at 21000 rpm (52000 × *g*) for 20 min at 35°C. The supernatant, being the nuclear sap or interchromosomal material, was collected preparatively with a yellow pipette tip and transferred to another tube. The supernatant was precipitated with 0.6 volume of isopropanol from 0.3 M NaAc. The lenticular precipitate, representing the chromosome fraction, was (1) supplemented with 100 μl of water and left to swell for 60 min before electrophoresis; (2) supplemented with 500 μl of water and left to swell for 10 min before precipitation with isopropanol; or (3) dissolved in the buffer containing 8 M urea and 8% glyoxal (denaturing conditions).

All the procedures were performed at 0°C. All the samples upon treatment were immediately precipitated with 0.6 volume of isopropanol from 0.3 M NaAc in 25-ml conic tubes to centrifuge in a bucket rotor in a K23 centrifuge at 4500 rpm for 20 min. The precipitate was dissolved in (1) 100 μl of water or (2) denaturing buffer at 65°C for 60 min. The amount of labeled material was determined routinely in Eppendorf tubes put into counting vials with a 1209 RackBeta counter (Finland). The samples were fractionated in 0.7% agarose gel. The DNA of chromosome fraction in the experiments under native conditions was not dissolved completely to reduce DNA degradation connected with dissolution but just left over 60 min for swelling. The "jellyfish" of undissolved, swollen precipitate was loaded into the well of agarose gel. During electrophoresis, chromosome DNA remained at the start. In the case of denaturing conditions, solution of nucleic acid material was loaded onto agarose gel without prior dialysis. Upon electrophoresis, the agarose gel was dried on a plate under a flow of hot air. X-ray film was exposed to the gel was overnight or for a period depending on the amount of labeled material.

## Abbreviations

FITC – fluorescein isothiocyanate

M/SAR – Matrix-scaffold attachment region

## Competing interests

Authors are required to complete a declaration of competing interests. All competing interests that are declared will be listed at the end of published articles. Where an author gives no competing interests, the listing will read 'The author(s) declare that they have no competing interests'.

When completing your declaration, please consider the following questions:

Financial competing interests

• In the past five years have you received reimbursements, fees, funding, or salary from an organization that may in any way gain or lose financially from the publication of this manuscript, either now or in the future? Is such an organization financing this manuscript (including the article-processing charge)? If so, please specify.

The work was funded by OOO Panagen.

• Do you hold any stocks or shares in an organization that may in any way gain or lose financially from the publication of this manuscript, either now or in the future? If so, please specify.

No.

• Do you hold or are you currently applying for any patents relating to the content of the manuscript? Have you received reimbursements, fees, funding, or salary from an organization that holds or has applied for patents relating to the content of the manuscript? If so, please specify.

Yes. We received funding from OOO Panagen, which has applied for patents relating to the content of manuscript.

• Do you have any other financial competing interests? If so, please specify.

No.

Non-financial competing interests

Are there any non-financial competing interests (political, personal, religious, ideological, academic, intellectual, commercial or any other) to declare in relation to this manuscript? If so, please specify.

If you are unsure as to whether you, or one your co-authors, has a competing interest please discuss it with the editorial office.

The author(s) declare that they have no non-financial competing interests.

## Authors' contributions

Vladimir A. Rogachev – AB

Anastasia Likhacheva – AB

Oksana Vratskikh – JY

Lyudmila V. Mechetina – AB

Tamara E. Sebeleva – AB

Sergei S. Bogachev – FG

Leonid A. Yakubov – FG

Mikhail A. Shurdov – FG
